# Performance analysis of automated evaluation of antinuclear antibody indirect immunofluorescent tests in a routine setting

**DOI:** 10.1007/s13317-018-0108-y

**Published:** 2018-09-21

**Authors:** Joyce J. B. C. van Beers, Melanie Hahn, Johanna Fraune, Kathleen Mallet, Christopher Krause, Wymke Hormann, Kai Fechner, Jan G. M. C. Damoiseaux

**Affiliations:** 1 Central Diagnostic Laboratory, Maastricht University Medical Center, P. Debyelaan 25, 6229 HX Maastricht, The Netherlands; 2 Institute for Experimental Immunology, EUROIMMUN Medizinische Labordiagnostika AG, Seekamp 31, 23560 Lübeck, Germany

**Keywords:** Autoantibodies, Antinuclear antibodies (ANA), Automation, Indirect immunofluorescence (IIF), Computer-aided microscopy

## Abstract

**Purpose:**

Indirect immunofluorescence (IIF) on the human epithelial cell-line HEp-2 (or derivatives) serves as the gold standard in antinuclear antibody (ANA) screening. IIF, and its evaluation, is a labor-intensive method, making ANA testing a major challenge for present clinical laboratories. Nowadays, several automated ANA pattern recognition systems are on the market. In the current study, the EUROPattern Suite is evaluated for its use in daily practice in a routine setting.

**Methods:**

A total of 1033 consecutive routine samples was used to screen for ANA. Results (positive/negative ANA screening, pattern identification and titer) were compared between software-generated results (EUROPattern) and visual interpretation (observer) of automatically acquired digital images.

**Results:**

Considering the visual interpretation as reference, a relative sensitivity of 99.3% and a relative specificity of 88.9% were obtained for negative and positive discrimination by the software (EPa). A good agreement between visual and software-based interpretation was observed with respect to pattern recognition (mean kappa: for 7 patterns: 0.7). Interestingly, EPa software distinguished more patterns per positive sample than the observer (on average 1.5 and 1.2, respectively). Finally, a concordance of 99.3% was observed within the range of 1 titer step difference between EPa and observer.

**Conclusions:**

The ANA IIF results reported by the EPa software are in very good agreement with the results reported by the observer with respect to being negative/positive, pattern recognition and titer, making automated ANA IIF evaluation an objective and time-efficient tool for routine testing.

**Electronic supplementary material:**

The online version of this article (10.1007/s13317-018-0108-y) contains supplementary material, which is available to authorized users.

## Introduction

Antinuclear antibodies (ANA) are well-appreciated biomarkers in the laboratory diagnostics of systemic autoimmune rheumatic diseases (SARD) [1]. Indirect immunofluorescence (IIF) testing still serves as the gold standard for ANA screening. IIF is performed on human epithelial cells (HEp-2 cells and its derivates) which allows differentiation of diverse fluorescence patterns and determination of antibody titers.

HEp-2 cells represent a broad spectrum of clinically relevant autoantigens, including those which have not been identified yet and/or may not be present as purified antigen in alternative, solid-phase (multiplex) assays [2]. The current international consensus on ANA patterns (ICAP) distinguishes between 29 different nuclear, cytoplasmic and mitotic fluorescence patterns (AC-1 till AC-29). With respect to HEp-2 cell positivity described in different international criteria, only the nuclear patterns are considered true ANA positive [3, 4]. In addition, a ‘negative’ pattern has also been defined (AC-0). Many of these fluorescence patterns can be assigned to relevant autoantibodies, due to the specific localization of the corresponding antigens, and may provide initial indications to the associated autoimmune disease [2]. Due to its low specificity (but high sensitivity), IIF needs to be followed by monospecific assays to identify autoantibody specificity [3]. If ANA is positive, titration of positive samples is also considered to be important because higher ANA titers are associated with a higher probability of SARD, while low ANA titers may also occur in apparently healthy individuals [5]. Also, the chance of identifying the specifically targeted antigen in ANA IIF using monospecific assays increases with the titer heights [6, 7]. Finally, minor patterns in mixed patterns often become visible only at higher dilutions.

Over the last decades, the number of ANA requests has considerably increased. Since IIF, and especially its evaluation, is a labor-intensive method, the high demand for ANA testing is a major challenge for present clinical laboratories. Automated platforms for computer-aided immunofluorescence microscopy, which have been introduced over the last 10 years, facilitate and standardize IIF evaluation and may help to solve this problem [8–10]. One of these platforms is the EUROPattern Suite consisting of an automated microscope and different classification software modules for evaluation of acquired digital images in ANA and ANCA (anti-neutrophil cytoplasm antibody) diagnostics as well as of Crithidia luciliae indirect immunofluorescence test (CLIFT) and different cell-based assays [11–15]. In ANA diagnostics, the system provides differentiation of negative/positive results, discrimination of various single and mixed nuclear, cytoplasmic and mitotic patterns and titer prediction. Results are suggested to the operator, accompanied by a calculated confidence value, and have to be verified by mouse click before being included into the patient history.

In the current study, we have evaluated the performance of the EUROPattern Suite (EPa) for ANA detection in daily clinical practice by comparing its automatically generated results (negative/positive, pattern recognition and titer) to the results obtained by visual evaluation of the digital images.

## Materials and methods

### Serum samples

Consecutive serum samples with ANA request were prospectively analyzed at the Central Diagnostic Laboratory of the Maastricht University Medical Center in Maastricht, the Netherlands, over a period of 6 months (November 2015–April 2016). 1098 samples were eligible for this study. Reasons for exclusion were too little serum available (*n* = 40) or technical artifacts (*n* = 25). Overall, 1033 samples were available for analysis.

### Automated ANA detection

Samples were analyzed on HEp-20-10 slides in a 1/100 dilution (Euroimmun, Lübeck, Germany). The assay was conducted according to manufacturer’s instructions. The cut-off decision for positivity is an antibody titer of 1/100. If positive, samples were titrated (1/320 and 1/1000). Images of the incubated slides were automatically acquired by the EUROPattern microscope (Euroimmun, Germany) and digitally transferred into the software for further evaluation. Images were interpreted, once automatically by the EPa software (Euroimmun, Lübeck, Germany) and, in a parallel approach, visually by two independent individuals (Observer 1 and 2). IIF images were evaluated with respect to negativity/positivity, pattern (homogeneous, speckled, nucleolar, centromere, nuclear dot, nuclear membrane, cytoplasmic) and titer (1:100; 1:320; 1:1000; > 1:1000).

### EUROPattern Suite

Images acquired by the microscope were automatically evaluated by the EUROPattern classification software. EUROPattern Suite (Euroimmun, Lübeck, Germany) consists of an automated microscope and sophisticated classification software for negative/positive discrimination, pattern recognition (accompanied by a confidence value) and titer calculation in ANA IIF. The steps of the automated immunofluorescence analysis process are carried out in a sequential order comprising the taking of the slides from a magazine, positioning of the slide and autofocusing, acquisition and subsequent processing of digital images, classification of the cells and quality control, pattern recognition and titer calculation of positive cells and finally merging of individual results into one report per sample. One image is taken per Biochip and is analyzed within 12 s. Software-proposed results are presented to the operator at the computer screen who has to validate them by a mouse click—individually for positive samples and batch wise for negative samples. Because the graphical user interface is an integral part of the laboratory management software EUROLabOffice (Euroimmun, Lübeck, Germany), the final results can be automatically transmitted to the laboratory information system (LIS) after confirmation and archiving it in the patient’s history [13].

The EUROPattern is configured with standard settings at default. The software, however, can be adjusted to specific requirements of a laboratory to better match with the established visual reading. In the current study, primary results were obtained by the software at default setting (unadjusted results). These data were used as basis for the optimization of the software with respect to the visual reading by the observer (adjusted results).

### Statistics

Relative sensitivity was calculated as the number of true-positive samples divided by the sum of true-positive and false-negative samples (times 100 to express in %); relative specificity was calculated as the number of true-negative samples divided by the sum of true-negative and false-positive samples (times 100 to express in %). True positive and true negative is defined by the respective reference method as indicated. Cohen’s kappa coefficient (*к*) was used to determine agreement between methods, because it takes into account the possibility of an agreement occurring by chance [16]. According to Landis and Koch, *к* < 0 means no agreement, *к* between 0 and 0.2 means little agreement, *к* between 0.21 and 0.40 means small agreement, *к* between 0.41 and 0.60 means moderate agreement, *к* between 0.61 and 0.80 means good agreement and *к* between 0.81 and 1 means almost perfect agreement [17].

## Results

### Negative/positive discrimination

Results obtained with the default software classifier, i.e., before adjustment, are summarized in supplementary Table 1. When considering either observer 1 or observer 2 as references, there was good agreement between with the EPa results and the visual readings, revealing *к* values of 0.64 and 0.66, respectively. As mentioned, the EPa software is flexible and settings were optimized/customized. Results obtained with the adjusted classifier are summarized in Table 1. The obtained *к* values for agreement between adjusted automatic and visual evaluation significantly increased to 0.81 and 0.79 for observer 1 and observer 2, respectively. The agreement improved because EPa software strongly reduced the number of “false”-positive results as also reflected in the increase in relative specificity (observer 1: 70.7–88.9%; observer 2: 82.7–93.2%), while hardly affecting relative sensitivity (observer 1: 96.4–99.3%; observer 2: 85.6–85.1%). Thus, the EPa software reached a very high relative sensitivity (99.3%) when compared to observer 1 (Table 1 and Supplementary Table 1). This implies that results reported negative by the classifier were also considered negative by observer 1. Interestingly, a similar level of agreement was observed between observer 1 and 2 with a к of 0.77 (supplementary Table 2).

**Table 1:** Comparison of software-generated and visual positive/negative classification

	Visual evaluation (observer 1)			Visual evaluation (observer 2)		
	Negative	Positive	Total (*N*)	Negative	Positive	Total (*N*)
EUROPattern (EPa) software (adjusted)						
Negative	673	2	675	620	55	675
Positive	84	274	358	45	313	358
Total (*N*)	757	276	1033	665	368	1033
Kappa agreement	0.81			0.79		
Relative sensitivity* (%)	99.3			85.1		
Relative specificity* (%)	88.9			93.2		

*To calculate relative sensitivity and specificity, the visual evaluation made by the observer (1 or 2) is regarded as correct

### HEp-2/IIF pattern assignment

As indicated above, the primary software classifier of EPa identified 430 samples as positive, while after adjustment only 358 samples were reported positive by the system (Table 1, Supplementary Table 1).This adjustment also affected the prevalence of false-positive ANA pattern assignments (Supplementary Table 3). Adjustment strongly reduced the number of false-positive nucleolar ANA (140 to 20), while the number of false-positive speckled ANA remained the same (89 to 90). False-positive homogeneous ANA, however, increased in number (43 to 80). The majority of these false-positive ANA (*n* = 54) was only positive in the 1/100 screening dilution. EPa-generated confidence values for those false-positive samples which exclusively showed a homogeneous pattern were examined (0.86 ± 0.07), but it did not differ significantly from the confidence values (0.93 ± 0.05) of the true-positive, solely homogeneous ANA with a 1/100 titer (*n* = 68).

The adjusted EPa software identified 358 samples as positive, and for 349 samples one or more ANA patterns were defined. Of the 276 positive samples, observer 1 identified 268 with at least one pattern. Hence, the EPa software, as well as observer 1 could not identify a pattern in a small subset of the samples (9 and 8, respectively) (Table 2). In both evaluation methods the majority of ANA positive samples showed only a single fluorescent pattern, i.e., 65.3% and 75.4%, respectively. As illustrated in Table 2, the EPa software identified more patterns per positive sample (1.5) than observer 1 (1.2).

**Table 2:** Pattern assignments

Number of patterns identified	Visual evaluation (observer 1), *n* = 276	EUROPattern (adjusted) (EPa) software, *n* = 358
0	8 (2.8%)	9 (2.5%)
1	202 (75.4%)	227 (65.3%)
2	61 (22.8%)	83 (23.5%)
3	5 (1.8%)	32 (9.2%)
4	0	6 (1.7%)
5	0	1 (0.3%)

Comparison of (adjusted) software-generated pattern assignments with visual interpretation by observer 1 is summarized in Table 3. Almost perfect agreement was achieved for the centromere (*к* = 0.93) and the cytoplasmic (*к* = 0.83) pattern. For the homogeneous (*к* = 0.71) and nucleolar (*к* = 0.74) pattern the agreement was good. This also holds for the nuclear dots pattern (*к* = 0.66), but the number of samples revealing this pattern by any of the two methods was very low. Agreement for the speckled (*к* = 0.49) and nuclear membrane (*к* = 0.57) was only moderate. As for the nuclear dots pattern, the number of samples that were assigned to the nuclear membrane pattern was also very low.

**Table 3:** Comparison of software-generated and visual pattern identification

	Visual evaluation (observer 1)						
	H	S	N	C	D	NM	Cyt
	*n* = 157	*n* = 63	*n* = 37	*n* = 16	*n* = 5	*n* = 2	*n* = 59
EUROPattern (adjusted) (EPa) software							
TP	147	55	34	15	4	2	51
FP	80	90	20	1	4	3	11
TN	796	880	976	1016	1024	1028	963
FN	10	8	3	1	1	0	8
Relative sensitivity (%)	93.6	87.3	91.9	93.8	80.0	100.0	86.4
Relative specificity (%)	90.9	90.7	98.0	99.9	99.6	99.7	98.9
Kappa agreement	0.71	0.49	0.74	0.94	0.61	0.57	0.83

*H* homogeneous, *S* speckled, *N* nucleolar, *C* centromere, *D* nuclear dots, *NM* nuclear membrane, *Cyt* cytoplasmic, *TP* true positive, *FP* false positive, *TN* true negative, *FN* false negative, observer 1 is considered the reference method

### Titer assignments

Titers determined by the observer 1 and EPa software were compared for the samples with a homogenous (*n* = 147) fluorescence pattern for which software and visual pattern evaluations were in agreement. In 120 samples (81.6%) with a homogeneous fluorescence pattern, the titers reported by both methods were similar. Accepting differences of ± one titer step, titers in 145 samples (98.6%) were concordantly assessed (Table 4).

**Table 4:** Titer estimation for homogeneous fluorescence pattern

Homogeneous (*n* = 147)	Visual evaluation (observer 1)			
	1/100	1/320	1/1000	>1/1000
EUROPattern (adjusted) (EPa) software				
1/100	**87**	*3*	1	0
1/320	*4*	**19**	*6*	0
1/1000	1	*9*	**13**	*0*
> 1/1000	0	0	*3*	**1**
Same titer (*n* = 120)				
Overall concordance	81.6%			
Difference of ≤ 1 titer step (*n* = 145)				
Overall concordance	98.6%			

Bold values indicate 100% agreement between visual observation and EUROPattern software. Italic values indicate one titer step difference between visual observation and EUROPattern software

## Discussion

Current clinical laboratories performing ANA IIF tests increasingly rely on automated workflows to standardize and accelerate the handling of the daily requests for ANA testing. Since particularly the evaluation of IIF tests is a time-consuming and error-prone step due to the subjectivity of the read out, many efforts have been undertaken into the development of platforms for computer-aided immunofluorescence microscopy. The technology is based on the automated image acquisition of the slides and subsequent evaluation of digital images with the help of classification software. The software is able to discriminate between negative and positive results and, depending on the system, between different HEp-2/IIF patterns and to calculate corresponding titers [18–23].

In this prospective study usability of the EUROPattern Suite (Euroimmun, Lübeck, Germany) for automated ANA evaluation in a routine setting was studied. The system provides positive/negative discrimination, recognition of various ANA patterns and titer calculation. Compared to visual positive/negative discrimination by observer 1, automated results generated by EUROPattern were in good agreement (*κ* = 0.78). A comparable concordance of results with *κ* = 0.77 was also noted for two independent observers. The automated system exhibited a very high relative sensitivity (99.3%) ensuring a high reliability on negative classification of samples. Those negative classified samples can be verified batch wise by the operator with a single mouse click, a feature that significantly accelerates the IIF evaluation process. On the other hand, a substantial number of “false”-positive results were obtained (relative specificity 88.9%). The majority of these samples were only positive in the 1/100 screening dilution. Previously, it has been shown for the automated evaluation of CLIFT that true-positive results given by the EUROPattern Suite reveal significantly higher confidence values than false-positive results [15]. Thus, the confidence value can be an efficient indicator for the reliability of a software-generated result which may support the operator during the verification process. However, in case of the examined low titer homogeneous positive samples, no significant difference in confidence values was observed between true- and false-positive results. The clinical relevance of low titer ANA is usually limited as these are often detected in healthy individuals and elderly without any clinical signs of SARD [9, 24, 25]. In addition, it is unlikely that an antigen specificity will be identified in these samples [24, 26, 27].

The visual interpreted results (observer 1) of the EPa compatible HEp-20-10 substrate were also compared to routine ANA testing on HEp-2000 cells (data not shown). Keeping in mind that different assay conditions are compared, still an ~ 10% increase of ANA positive results was observed (compared to a decrease of < 1% ANA positive results). This elevated positivity rate may lead to additional costs for follow-up testing (i.e., anti-dsDNA and anti-ENA antibodies) when implementing the automated platform. On the other hand, it can also be argued that these additional positive results enable case finding for establishing an early diagnosis and preventing severe complications [28].

Several studies on automatic ANA pattern recognition have already been published [8, 9, 29–31]. Some of these studies, however, compared two different assays using different substrates and different screening dilutions, making it difficult to evaluate the automatically generated ANA results [29, 32]. Melegari et al. evaluated the Aklides system (Medipan GmbH, Dahlewitz/Berlin, Germany). In their set-up, the same conditions were met in automated and visual ANA positive/negative evaluation (same substrate, same manufacturer, same dilutions), but in visual microscopy a traditional fluorescence microscope was used. They detected a similar percentage of discrepant samples (7%) as in the current study (9%), but lower relative sensitivity (95%) and specificity (82%) for the Aklides system [33], [34]. Voigt et al. used a similar experimental design as in our current study and compared automatically generated results (EPa) and visual interpretation of digital images. As such, the HEp-2 cellular substrate, serum dilutions, and also digital images were identical. However, the study was performed retrospectively and the sample size was smaller than the one used in the current setting, which could explain the higher agreement and higher relative sensitivity and specificity observed in their study [11].

Bizzaro et al. evaluated six different platforms (including EUROPattern) by comparing the automatically generated ANA results for 126 pre-characterized samples (92 ANA positive and 34 ANA negative samples). In this study, sensitivities ranged between 93.5% and 98.9% with EUROPattern revealing a sensitivity of 96.7%. Differentiating seven different ANA patterns, however, EUROPattern was the system recognizing the highest number of patterns and in best agreement with the pre-characterization (79%). The EUROPattern software differs from the other automated platforms for computer-aided immunofluorescence microscopy in being a flexible/adjustable system. Indeed, software adjustment strongly reduced false-positive overall results, leading to an improved agreement with visual reading. With respect to pattern identification, the adjustment revealed divers effects, reducing the number of false-positive determinations of the nucleolar pattern but increasing the number of false-positive homogeneous samples. Although not part of this study, it is noteworthy that a more recent, second software adjustment achieved a strong reduction in the false-positive homogeneous pattern classification (back to 54 false-positive results), without affecting any of the other evaluation characteristics (data not shown). While computer-aided immunofluorescence microscopy is designed to facilitate the workflow of IIF, it also entails the possibility for harmonization of IIF result interpretations. Obviously, adjusting the EPa software to accommodate for the wishes of the user will hamper the latter goal. As such, it is interesting to see that the adjustments not only improved agreement with observer 1, but to a similar extend also improved agreement with observer 2, being an employee of Euroimmun. This observation, at least, suggests that harmonization remains feasible and that software adjustments may improve the system for multiple users over the forthcoming years.

## Conclusion

Our study shows that automatically generated ANA results reported by EPa have an overall good agreement with visual interpretation of the digital images with respect to negativity/positivity, pattern recognition, and titer. The EPa software is even better in recognizing mixed patterns than visual evaluation. This makes automated evaluation of ANA IIF suitable for the routine setting. Challenges lie in the further expansion of the pattern spectrum that can be identified with the EPa software (e.g., the dense fine speckled pattern) and the further accommodation to the ICAP classification [2].

Automated evaluation of the HEp-2/IIF test is a helpful tool in the laboratory to counter the raising workload as a result of the increasing number of ANA testing requests, and, eventually, may be an important tool in further harmonization of ANA diagnostics.

## Electronic supplementary material

Below is the link to the electronic supplementary material.

Supplementary material 1 (PDF 24 kb)
