# Supplementary material for: Performance analysis of automated evaluation of antinuclear antibody indirect immunofluorescent tests in a routine setting
Source: Auto Immun Highlights. 2018 Sep 21;9(1):8. doi: 10.1007/s13317-018-0108-y (PMC6147779; doi:10.1007/s13317-018-0108-y)
Supplement: Supplementary file 1 — Supplementary material 1 (PDF 24 kb) [file 13317_2018_108_MOESM1_ESM.pdf]

Supplementary data

Supplementary Table 1. Comparison of non-adjusted software-generated and visual positive/negative classification.

Supplementary Table 2. Comparison of visual positive/negative classification between the two users.

Supplementary Table 3. Comparison of non-adjusted software-generated ANA pattern classification.

Supplementary Table 1. Comparison of non-adjusted software-generated and visual positive/negative classification.

		Visual evaluation observer 1			Visual evaluation observer 2		
		Negative	Positive	Total (N)	Negative	Positive	Total (N)
EUROPattern (EPa)Software not adjusted	Negative	593	10	603	550	53	603
	Positive	164	266	430	115	315	430
	Total (N)	757	276	1033	665	368	1033
	Kappa agreement	0.64			0.66		
	Relative sensitivity* (%)	96.4			85.6		
	Relative Specificity* (%)	70.7			82.7		

*In order to calculate relative sensitivity and specificity, observer (1 or 2) is considered the reference method.

Supplementary Table 2. Comparison of visual positive/negative classification between the two users.

		Visual evaluation observer 1		
		Negative	Positive	Total (N)
Visual evaluation observer 2	Negative	660	5	665
	Positive	97	271	368
	Total (N)	757	276	1033
	Kappa agreement	0.77		
	Relative Sensitivity* (%)	98.2		
	Relative Specificity* (%)	87.2		

*In order to calculate relative sensitivity and specificity, observer 1 is considered the reference method.

Supplementary Table 3. Comparison of non-adjusted software-generated ANA pattern classification.

	Homogeneous		Speckled		Nucleolar	
	Unadjusted	Adjusted	Unadjusted	Adjusted	Unadjusted	Adjusted
TP	143	147	53	55	37	34
**FP**	**43**	**80**	**89**	**90**	**140**	**20**
TN	833	796	881	880	856	976
FN	14	10	10	8	0	3

TP = true positive; FP = false positive; TN = true negative; FN = false negative; observer 1 is considered the reference method.
